# Carbon Monoxide Poisoning With Concomitant Mucosal Injury and Chemical Pneumonitis Using Sulfuric and Formic Acids in a Self-Harm Attempt

**DOI:** 10.7759/cureus.17894

**Published:** 2021-09-11

**Authors:** Nicholas Lewis, Becky DeVore, LaDonna Gaines, Justin K Arnold

**Affiliations:** 1 Emergency Medicine, University of South Florida Morsani College of Medicine, Tampa, USA; 2 Alabama Poison Information Center, Children’s of Alabama, Birmingham, USA

**Keywords:** deliberate self harm, formic acid, sulfuric acid, toxicology, chemical pneumonitis, carbon monoxide poisoning

## Abstract

The intentional liberation of carbon monoxide through the dehydration of formic acid has been reported with increasing frequency in the literature as a method of self-harm. Online forums have popularized this method of self-harm due to the ease of access of the required reagents, as well as the ability to perform the reaction under ambient conditions. The basis of this method of suicide is the use of sulfuric acid as a dehydrating agent, leading to the decomposition of formic acid into carbon monoxide gas. In addition to the exposure to carbon monoxide liberated by this reaction, the relatively high vapor pressure of formic acid can inadvertently lead to its inhalation and subsequently cause damage to the aerodigestive tract. We report a 21-year-old male who presented with manifestations of acute carbon monoxide poisoning and concomitant chemical pneumonitis. Increased awareness and understanding of this method of self-harm is critical in ensuring appropriate precautions are taken when caring for these individuals.

## Introduction

This article was previously presented as a meeting abstract at the 2017 North American Congress of Clinical Toxicology Meeting on October 14, 2017.

Carbon monoxide (CO) is a colorless and odorless gas that is most commonly produced following the incomplete combustion of fossil fuels. Its ubiquitous presence associated with the use of generators, heaters, stoves, and grills, as well as its lack of distinguishing warning features, has led to it consistently being one of the deadliest sources of non-medicinal poisoning in the U.S. 24,890 total deaths by CO poisoning were reported nationwide from 1999 to 2014, excluding those instigated by exposure to outdoor fires [1]. Of these deaths, 73.2% (18,231) were considered intentional poisonings [1]. Regulations such as the widespread adoption of the catalytic converter and statewide CO monitor mandates, coupled with improved awareness of the risks posed by the gas, has led to a decrease in the number of reported deaths from 1,874 total deaths/year in 1999 to 1,245 deaths/year in 2014 [1].

The complete and exact mechanism underlying the toxicity of CO exposure has not yet been entirely elucidated [2]. Following its absorption through pulmonary capillaries, CO preferentially binds the heme moiety of hemoglobin, promoting an allosteric change that inhibits oxygen offloading and ultimately leads to global hypoxia [3]. The degree by which carboxyhemoglobin (COHb) levels are elevated does not reliably predict patient presentations or outcomes, suggesting additional mechanisms may be in part responsible [2]. Proposed mechanisms include, but are not limited to, a leftward shift in the oxygen dissociation curve [2], generation of reactive species including peroxynitrite with subsequent oxidative stress [2,4], and heterotypic aggregation between platelets and neutrophils [5].

There is a great deal of variability in the clinical presentation underlying CO poisoning. Non-specific symptoms in mild inhalations - including headaches, fatigue, and nausea - can lead to the misdiagnosis of CO poisoning for acute viral syndromes [6]. Patients can additionally present with variable neurological symptoms (including altered mental status, seizures, syncope, and coma), cherry-red skin, and cardiovascular symptoms that are likely secondary to ischemia [7]. While baseline COHb concentrations can vary based on tobacco smoking status and environmental pollutants, concentrations above 3%-4% in nonsmokers can be used to diagnose the condition when used in conjunction with a suspected history [3]. In heavy smokers, baseline COHb concentrations can be as high as 15% [3]. Hyperbaric oxygen therapy has been used as adjunctive therapy in some patients after CO poisoning; however, methodological limitations have prevented definitive guidelines from being established [3].

An alternative method of intentional CO poisoning has been described with increasing frequency in the literature, largely in part due to its discussion on online forums. CO can be intentionally liberated following a chemical reaction between sulfuric acid and formic acid [8]. Herein, we present a 21-year-old male who presented to the emergency department with CO poisoning, hemoptysis, pulmonary edema, and chemical pneumonitis following exposure to CO produced from a mixture of formic and sulfuric acids.

## Case presentation

A 21-year-old male with no significant medical history was found unconscious at his university residence approximately one hour after a suicide attempt. A large bowl containing an unknown solution was found on the scene, which was later identified as a mixture of sulfuric acid and formic acid. The patient reported later he purchased the individual components after learning about the suicidal method in an online forum. He reported mixing the chemicals together in the bowl, placing a towel over his head and the bowl, and leaning over it to inhale the CO by-product.

Upon arrival to the emergency department (ED), he was awake and alert, demonstrating frank hemoptysis associated with the chest and abdominal pain. Rhonchi were noted on his lung exam. His initial vital signs included a blood pressure of 110/73 mmHg, heart rate of 126 bpm, respiratory rate of 32 bpm, pulse oximeter oxygen saturation 92% on room air, and a temperature of 101.4 °F. He was immediately placed on a non-rebreather mask with 100% FiO_2_.

His laboratory evaluation was notable for a WBC of 23.37 mcL, BUN of 25 mg/dL, and a creatinine of 1.5 mg/dL. His arterial blood gas at presentation showed a pH of 7.489, a pCO_2_ of 28.9 mmHg, a pO_2_ of 53 mmHg, an HCO_3_ of 29.1 mmol/L, and a COHb of 27.4%. A radiologist reviewed his chest x-ray and reported findings consistent with pulmonary edema and chemical pneumonitis.

The patient was endotracheally intubated within 30 minutes of arrival to ED due to oxygen desaturation and persistent hemoptysis. Levofloxacin and methylprednisone were both administered. Serial arterial blood gases with co-oximetry showed a decrease in his COHb to 3.3% and 1.6% at two and 25 hours after initial presentation, respectively.

Fortunately, the patient was extubated 21 hours after his initial exposure. He developed mild rhabdomyolysis (peak CPK 1,781 µg/L) and oliguria, both of which resolved with IV fluids and supportive care by day of discharge.

## Discussion

**Figure 1:**
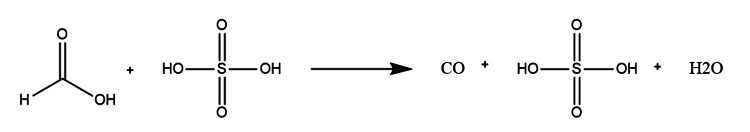
Reaction scheme detailing the dehydration of formic acid by sulfuric acid, yielding carbon monoxide and hydrated sulfuric acid.

We report a suicidal patient who survived and made a full recovery after combining formic and sulfuric acids to produce CO resulting in severe toxicity. This case, unfortunately, represents one instance of an increasingly common method by which individuals have used this dehydration reaction to liberate CO with the suicidal intention [9-15]. While the mixture of sulfuric acid and formic acid has not historically seen much use as a method of self-harm, it has been employed with increasing frequency due to its popularization by online websites and forums. This reaction is commonly employed in laboratory settings as a means to produce CO. Sulfuric acid acts as a dehydrating agent to decompose formic acid into CO and hydrated sulfuric acid (Figure 1) [8]. Despite formic acid not being a common household chemical, it is used for purposes related to agriculture and manufacturing and is readily available for purchase. Similarly, sulfuric acid can be purchased from several online vendors, primarily for use as a drain cleaner. The ease of access of these two reagents and the ability to perform this reaction quickly under ambient conditions is in part responsible for increased employment of this method.

While exposure to CO was the primary precipitator of the patient’s presentation, it should also be noted that the chemical pneumonitis and damage to the aerodigestive tract may have been secondary to exposure to vaporized formic acid components. Cutaneous exposure to formic acid can initially present with caustic topical injuries, while ingestion of the acid can damage the pharyngeal mucosa and be absorbed, ultimately contributing to metabolic acidosis, intravascular hemolysis, hemoglobinuria, and renal failure [16]. Most relevant to this case, however, is the inhalation of formic acid vapors. Since formic acid has a vapor pressure of approximately 43 mmHg, it readily forms vapors that can be inhaled [17]. Exposure to these vapors can lead to chemical pneumonitis, with presentations that can include symptoms such as dyspnea, cough, and erythematous inflammation of the respiratory tract [16]. While sulfuric acid has an insignificant vapor pressure (0.001 mmHg), damage to the lungs is still possible through the inhalation of small droplets [18]. Additionally, the risk of cutaneous exposure or exposure through accidental ingestion exists for both sulfuric acid and formic acid.

Since CO is odorless and colorless, it is impossible to detect in an environment without the use of CO monitors. As a result, first responders who are not familiar with this reaction are at risk for exposure to CO and caustic injury, as reported by Yang et al. in which a father attempted to perform cardiopulmonary resuscitation on his son who had employed this method [11]. It is essential that efforts are made to raise awareness about this method of suicide to first responders and emergency providers to assure appropriate precautions are taken when caring for these individuals.

Since those who present with CO poisoning may show non-specific and variable symptoms, exposure to the compound via this method may not be apparent and treatment may be delayed. It is thus imperative that clinicians learn to recognize components of the clinical presentation that are indicative of this method of suicide. In particular, specialists in poison information (SPIs) and poison control centers should be sure to determine if there was an exposure to any other strong acid, such as formic acid or sulfuric acid, or a mixture of the two. This will help determine if there was possible exposure to CO and guide management of patients, any nearby individuals, and first responders who may be affected by both CO and possibly pulmonary caustic fumes.

## Conclusions

The preceding case describes an increasingly common method by which individuals dehydrate formic acid to produce CO with suicidal intention. This method of generating CO places patients at higher risk for concomitant chemical pneumonitis, which may introduce further challenges in their management. It is pertinent for healthcare providers to develop an awareness of this method of suicide in order to afford patients quicker access to appropriate treatment. Additionally, a more universal understanding will allow first responders to take appropriate safeguards and minimize the risk that they take in caring for these individuals.
